# Passing the Stomach Tube

**Published:** 1902-01-18

**Authors:** 


### Hospital Clinics and Medical Progress.

### PASSING THE STOMACH TUBE.

So frequent are disorders of the stomach and so large a part of the work of the general practitioner consists of attempts, more or less unsuccessful, to unravel the intricacies of the symptoms met with and to ascertain their cause, that it is somewhat surprising that more use is not made of the test meal and the stomach tube with the object of discovering exactly how the organ is doing its work. This method of investigation is frequently enough employed in hospital practice, and is in constant use by certain specialists, but we undertake to say that in general practice it is much less employed than it might be, and that even in consulting practice it is far too often the case that an authoritative diagnosis, with the inevitable prescription, is given without any complete investigation of the case having been made, if by the term "complete" we mean an investigation which includes every known means of ascertaining the exact condition of affairs. This being so we may usefully refer to a recent paper by Dr. Mark J. Knapp [1] of New York, in which he describes a procedure for introducing the stomach tube which differs somewhat from that taught in some of the text-books. He first of all asks, Are there any objections and contra-indications to the use of the tube? and he throws scorn on the contra indications given in the text-books where, he says, almost anything is regarded as an objection—disease of the heart, the lungs, the aorta, pregnancy, etc. But why, he asks, should a soft-rubber tube not be allowed to pass the œsophagus while a bit of bread with its ragged and pointed ends is granted this precious privilege? Do we not know from our successful experience with the soft-rubber tube in other parts of the body that it has been convincingly demonstrated that it is inoffensive and incapable of causing physical injury? "The very reason for using soft-rubber tubing is because it readily and meekly adapts itself to its surroundings, flattening itself whenever other progress is impossible, taking—possibly involuntarily—the only course open to it, twisting and turning whenever deemed wise to reach its destination, and stops short only when further progress is absolutely barred—qualifications and virtues that many of us might emulate with profit." A soft-rubber tube certainly cannot cut nor cause any abrasions, and if not hard—and this it should never be—it will readily yield to any pressure that any tumour, aneurysm not excepted, might cause." As a general rule, so long as the patient can swallow hard substances, such as meat and bread, without inconvenience, there exists no contraindication to the introduction of the tube.

If the regulation stomach tube be not at hand one can make one's own from ordinary soft-rubber tubing by cutting off the desired length, cutting the holes that are wanted and smoothing them with a red-hot glass rod, and washing off the sticky rubber with chloroform. The use of any directing rod within the lumen of the tube is to be condemned. The diameter of the tube may vary from "the size of an ordinary catheter" to about three-eighths of an inch, and it is well to have at hand two sizes, the smaller, which goes down the more easily, to be used on the first examination, and the larger to be used when the test meal is aspirated. The length of the tube should be about 28 inches, from 20 to 22 inches being the length between the incisor teeth and the cardia. It is a good plan to have the proximal or external end of the tube connected by a piece of the glass tubing to another piece of tubing, the glass portion enabling one to see the nature of the stomach contents as it passes out, so that if, in aspirating, blood should be noticed, the tube may be at once withdrawn. One need not be frightened about the blood, as it is caused in most instances by the patient straining; still one had better withdraw the tube.

As to the actual passing of the tube Dr. Knapp advises that we begin by explaining to the patient exactly what is going to be done and in what manner he can render assistance; and demonstrate to him the absolute impossibility of the tube doing any harm; and, especially, impress upon him the necessity of keeping on breathing. The tube is immersed in warm water, beyond which no other lubricant is necessary. False teeth must be removed. The patient is given a small basin which he is told to hold with both hands under his chin. This basin not only serves to catch any saliva which may flow, but also busies the hands of the patient, and keeps him from reaching for the tube.

The patient being now ready seated on an ordinary chair, the physician stands at his right side and puts his left arm round the back of the patient's neck, by doing which not only does he afford him some support but he has even the most robust patient under complete physical control. Then with our left arm round the back of the patient's neck, says Dr. Knapp, we hold the proximal or external end of the tube between the thumb and index finger of the left hand, the tube running through the palm of this hand, and the opening of the tube being thus kept away from us. This left hand is all the time kept close to the patient's mouth. The distal end of the tube—the part which goes into the stomach—is now taken between the thumb and index finger of the right hand, like a pen, and is introduced into the patient's mouth, which must not be opened any wider than is absolutely necessary, letting the tube pass all the time between the flexed third and fourth fingers of the left hand, by aid of which manœuvre the side motions of the tube are limited, and kinking is prevented. As soon as we believe the tube to have reached the back of the pharynx, we tell the patient to swallow and to keep on breathing deeply. The tube is now fed into the stomach, still passing between the third and fourth fingers of the left hand. It is not necessary to have the patient protrude his tongue or to put our finger into his mouth as a guide, as is sometimes taught.

This is the usual method of introducing the stomach-tube, and it very seldom fails. When such failure does occur, however, Knapps' stomach-tube director should be used, which is a guide by which the end of the stomach-tube is carried into the œsophagus, when it can then be pushed forwards into the stomach. The director dispenses with the co-operation of the patient, and is useful in children, nervous persons, and the insane, but in ordinary cases of stomach disorder is not required.

As soon as the tube has entered the stomach the aspirator ball must be at once taken in the right hand, compressed, and immediately attached to the tube which is still being held by the left hand. When the aspirator bag has filled itself, the tube must be at once withdrawn, still passing between the third and fourth fingers of the left hand by which the end of the tube must be caught and compressed before it leaves the mouth, otherwise there will be a mess. Having then got the sample in the aspirator bag, its condition must be examined.

From another American paper [2] we cull another "tip" for introducing the stomach tube, which is in one sense almost the exact opposite of the above. Instead of ensuring that the tube is soft and supple. Dr. Crenshaw freezes the tip, and so renders it stiff. This, however, is not the object of the freezing, the intention being to secure a slight temporary anæsthesia of the fauces and pharynx by means of the cold rubber. Dr. Crenshaw says that an effective and entirely safe method of preventing the nausea occasioned by the passage of the stomach tube is that of freezing two or three inches of the extremity of the tube just prior to its introduction. In this way the cold of the frozen rubber is applied just where anæsthesia is needed, and thus the irritability is overcome. The extremity of the tube may be readily frozen by a few moments' spraying with ethyl chloride. Other methods have been tried, but this has proved the most satisfactory.

[1] New York Medical News, Dec. 7. [2] New York Med. Rec., Dec. 21

